# Identification of essential genes in *Mycobacterium avium* subsp. *paratuberculosis* genome for persistence in dairy calves

**DOI:** 10.3389/fmicb.2022.994421

**Published:** 2022-10-20

**Authors:** Razieh Eshraghisamani, Amanda J. Mirto, Joyce Wang, Marcel A. Behr, Herman W. Barkema, Jeroen De Buck

**Affiliations:** ^1^Department of Production Animal Health, Faculty of Veterinary Medicine, University of Calgary, Calgary, AB, Canada; ^2^Environmental Health and Safety, University of Wisconsin-Madison, Madison, WI, United States; ^3^Department of Medicine, Faculty of Medicine, Health Centre, McGill University, Montréal, QC, Canada

**Keywords:** *Mycobacterium avium* subsp. *paratuberculosis*, transposon-insertion sequencing, mycobacterial pathogenesis, essential genes, cattle

## Abstract

To cause disease *Mycobacterium avium* subsp. *paratuberculosis* needs to enter mammalian cells, arrest phagosomal maturation and manipulate the host immune system. The genetic basis of the bacterial capacity to achieve these outcomes remains largely unknown. Identifying these genes would allow us to gain a deeper understanding of MAP’s pathogenesis and potentially develop a live attenuated Johne’s disease vaccine by knocking out these genes. MAP genes demonstrated to be essential for colonization in the natural host, ruminants, are unknown. Genome-wide transposon mutagenesis and high-throughput sequencing were combined to evaluate the essentiality of each coding region in the bacterial genome to survive in dairy calves. A saturated library of 3,852 MAP Tn mutants, with insertions in 56% of TA sites, interrupting 88% of genes, was created using a MycoMarT7 phagemid containing a mariner transposon. Six calves were inoculated with a high dose of a library of MAP mutants, 10^11^ CFUs, (input) at 2 weeks of age. Following 2 months of incubation, MAP cells were isolated from the ileum, jejunum, and their associated lymph nodes of calves, resulting in approximately 100,000 colonies grown on solid media across 6 animals (output). Targeted next-generation sequencing was used to identify the disrupted genes in all the mutants in the input pool and the output pool recovered from the tissues to identify *in vivo* essential genes. Statistical analysis for the determination of essential genes was performed by a Hidden Markov Model (HMM), categorizing genes into essential genes that are devoid of insertions and growth-defect genes whose disruption impairs the growth of the organism. Sequence analysis identified 430 *in vivo* essential and 260 *in vivo* growth-defect genes. Gene ontology enrichment analysis of the *in vivo* essential and growth-defect genes with the highest reduction in the tissues revealed a high representation of genes involved in metabolism and respiration, cell wall and cell processing, virulence, and information pathway processes. This study has systematically identified essential genes for the growth and persistence of MAP in the natural host body.

## Introduction

The dairy cattle industry around the world is affected by Johne’s disease (JD), a chronic intestinal inflammation in ruminants caused by *Mycobacterium avium* subsp. *paratuberculosis* (MAP). Johne’s disease is a production-limiting infection with clinical manifestation of chronic diarrhea, gradual weight loss, decreased milk production, reduced fertility and eventually death due to dehydration and cachexia. MAP is transmitted mainly through a fecal-oral route and while calves are likely infected early on in their life they can be infected with MAP at least up to 1 year of age with a higher infectious dose and longer exposure time (Mortier et al., 2013). MAP invasion is believed to happen mostly in the ileum and to a lesser degree in the distal jejunum (Ponnusamy et al., 2013; Arsenault et al., 2014). The mechanism by which MAP invades the tissue is primarily through the microfold cells of the Peyer’s patches although enterocytes may also play an important role in the internalization of MAP (Momotani et al., 1988; Pott et al., 2009).

The dairy industry suffers direct losses stemming from reduced milk production, early mortality, prematurely culling infected animals, and decreased carcass value (Ott et al., 1999; Chi et al., 2002). Even though JD in dairy cattle has been studied for over a century, prevention strategies have remained stagnant. Paratuberculosis control in sheep in Australia heavily relies on the use of a killed vaccine (GudairTM, Zoetis, Australia) that is reported to enhance both cell-mediated and humoral immunity. However, currently available vaccines for cattle are hindered by their shortcomings, including cross-reactivity with diagnostics for bovine tuberculosis, side effects evident as granulomas at the injection site, and only partial reduction of fecal shedding (Kalis et al., 2001; Köhler et al., 2001; Stabel et al., 2011). Previously, a MAP *relA* mutant was tested in bovine macrophages, as well as a goat and calf infection model. While the *relA* mutant was cleared from the calves, it did not prevent the colonization of the challenge strain (Park et al., 2011). All other live attenuated vaccine (LAV) candidates have been tested in goats and non-ruminant animals (Park et al., 2011; Hines et al., 2014). The present study aimed to test a large number of essential MAP mutants in cattle.

MAP has evolved to successfully infect ruminants and utilizes many different mechanisms to evade the immune system and dysregulate the host cells’ metabolism (Arsenault et al., 2014). Identifying genes that MAP requires to survive in its natural host will aid in identifying virulence mechanisms and in understanding MAP pathogenesis. This study will also identify genes that could be targeted in the development of live-attenuated vaccine strains.

**FIGURE 1:**
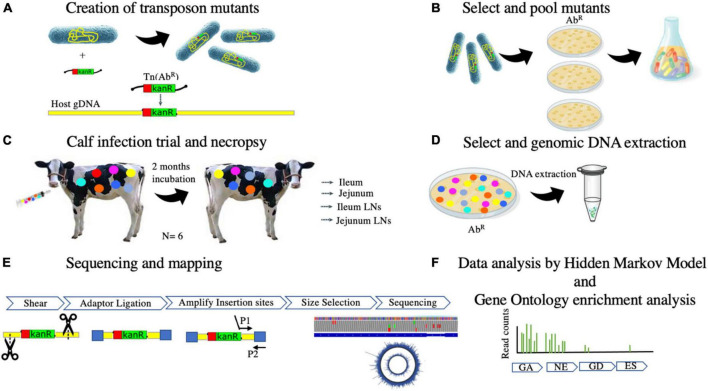
**(A–F)** Overview of the experimental procedures to identify essential genes for MAP to persist in natural ruminant host by transposon-sequencing.

Advancements in transposon-insertion sequencing methods have enabled a tracking method to identify essential genes for growth and persistence on a genome-wide scale in different genera of bacteria (Cain et al., 2020). Instead of evaluating the survival in an infection model of one MAP mutant strain at a time, Wang et al. used a method for evaluating thousands of MAP mutants concurrently using a mouse model (Wang et al., 2014). A library of mutants was developed using a MycoMarT7 phagemid containing a mariner transposon which inserts randomly at TA sites in the MAP genome creating disrupted genes (Siegrist and Rubin, 2009). The researchers were able to produce a library of mutants representing 56% of the genes in the MAP genome. Spleens were collected and targeted whole-genome sequencing was performed to evaluate disrupted virulent genes that were no longer present in the tissue (Wang et al., 2014). The aim of the present study was to evaluate *in vivo* essential MAP genes by determining mutant MAP survival in dairy calves. The use of dairy calves as the model for studying MAP survival ensures the discovery of genes required for establishing infection in the natural host. After generating a saturated library of mutants using a mariner transposon, thousands of potential mutants were studied congruently. Targeted next-generation sequencing revealed the disrupted virulence genes that were unable to survive in the host tissue (Figure 1). Identification of MAP essential genes could drive the development of live-attenuated or subunit vaccines capable of stimulating an immune response that prevents MAP from establishing an infection and subsequent fecal shedding, ultimately reducing the prevalence of JD.

## Results

### Generation of *mycobacterium avium* subsp. *paratuberculosis* mutant library and characterization of input and output pool

Total reads and colony count for the inoculum and each sample are presented in Table 1. Three mutant libraries were pooled and grown to a single inoculum (*n* = 1) which was aliquoted into 7 vials for 6 calves containing 10^11^ CFU of MAP mutants enumerated by the wet weigh method. One vial was used for sequencing and characterizing the inoculum. The inoculum was prepared from estimated 100,000 colonies grown on 7H11 plates and more than 8 million raw reads were obtained, mapping to 29,266 TA sites. 23,580 intragenic TA sites were interrupted. A total of 5,686 insertions were identified in the intergenic regions which accounts for 19% of the TA dinucleotide insertions in the inoculum.

**TABLE 1:** CFUs collected from all calf tissues and sum of reads per tissue from the targeted next-generation sequencing.

Animal	Tissue	Colonies collected/2.5 g of tissue	Total sequencing reads
Calf 1	Ileum	2,201	7,552,792
	Jejunum	2,643	8,316,242
	Ileum LN^1^	583	7,453,063
	Jejunum LN	911	8,369,757
Calf 2	Ileum	10,228	8,044,558
	Jejunum	2,162	7,972,065
	Ileum LN	425	7,311,431
	Jejunum LN	6,113	8,833,800
Calf 3	Ileum	27,024	6,683,118
	Jejunum	27,313	10,755,619
	Ileum LN	929	8,913,916
	Jejunum LN	1,649	11,777,854
Calf 4	Ileum	1,762	6,206,280
	Jejunum	5,527	8,696,110
	Ileum LN	4,298	7,600,686
	Jejunum LN	4,221	11,838,972
Calf 5	Ileum	1,178	7,316,934
	Jejunum	620	7,875,721
	Ileum LN	397	7,594,980
	Jejunum LN	2,465	13,393,191
Calf 6	Ileum	392	–
	Jejunum	383	–
	Ileum LN	17	–
	Jejunum LN	44	–
Control calf 1	Ileum	255	–
	Jejunum	3080	–
	Ileum LN	428	–
	Jejunum LN	0	–
Control calf 2	Ileum	2527	–
	Jejunum	0	–
	Ileum LN	196	–
	Jejunum LN	85	–
Inoculum	–	–	8,605,962

^1^LN, lymph nodes.

Targeted sequencing of the inoculum revealed that insertion mutations were present in 29,266/52,384 (56%) of all TA sites, corresponding to 3,852/4,350 (88%) of genes.

A variable number of colonies were recovered from approximately 10 g of tissues of the inoculated calves at 2 months post-inoculation, ranging from 397 to 27,313. One of the six calves provided less than 1,000 colonies over all 4 tissues and was eliminated from the analysis as it was considered to not represent an established infection. Not enough CFUs were recovered from each animal nor tissue. Although tissues were individually sequenced, to be able to represent the MAP genome and increase the power of analysis, all calves and tissue samples were combined, and 102,648 colonies were collected and sequenced.

### Identification of *in vivo* essential genes

**FIGURE 2:**
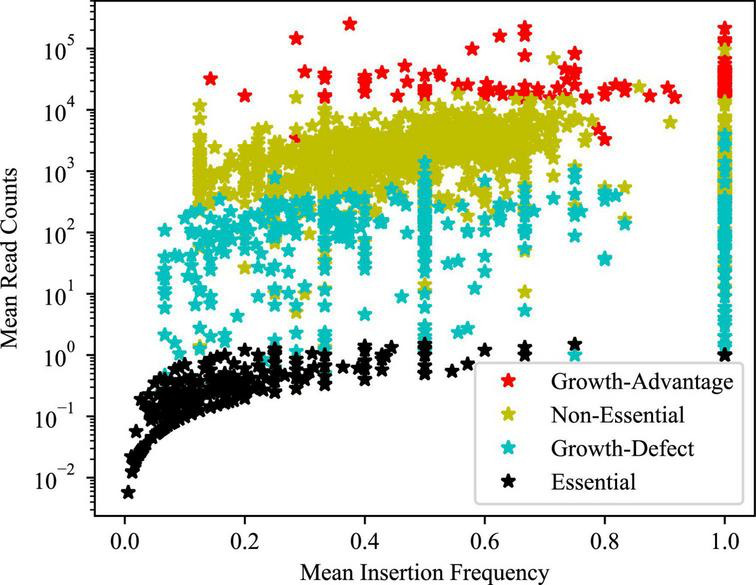
Mean insertion density and read counts for the regions identified by HMM. This graph illustrates the distribution of non-zero read counts and insertion frequencies for the regions identified by HMM that are defined as consecutive sites belonging to the same state.

A Hidden Markov Model (HMM) was applied to the sequencing data, where the sequence of observations was read counts at each TA site throughout the genome and each TA site is assumed to be in one of the four potential states: essential, growth-defect, non-essential, and growth-advantage (Figure 2 and Table 2). Essential genes are genes with low numbers of insertions, growth-defect genes whose disruption impairs the growth of the organism, non-essential genes whose insertion does not affect the growth of the organism, and growth-advantage genes that have metabolic costs and their insertion promote the growth of the organism. The state of adjacent genes has effects on each other state, differentiating between essential and non-essential genes as well as identifying genes whose disruption hinders or facilitates growth. A total of 430 and 260 genes were identified as *in vivo* essential and growth-defect genes, respectively (Supplementary Tables 1, 2, 8, 9 in Supplementary material 1). This included 169 genes with only a single TA mutant represented in the inoculum, 109 genes with 2 TA mutants, and 84 with 3 TA mutants and 328 genes with 4 or more TA mutants. Over 64% of orthologues of these MAP genes are also *in vitro* essential regions in *M. tuberculosis* H37Rv (Sassetti et al., 2003; Griffin et al., 2011; DeJesus et al., 2017).

**TABLE 2:** State identification statistics.

State of genes	Total% of genome	Mean read counts	Mean insertion density
Non-essential	73.3	3,403.9	0.521
Essential	16	0.1	0.068
Growth-defect	8.6	123	0.281
Growth-advantage	2.1	37,277.9	0.661

A measure of mean insertion density is the fraction of TA sites that contain at least one insertion averaged for each of the regions of a given state. Mean read counts are the mean value of non-zero read counts for each.

### Identification of *in vitro* essential genes

The inoculum represented 88% of the genes in the A1-157 genome. The remaining 12% of genes could be required for *in vitro* survival. Imperfect saturation of the library would result in an overestimation of these *in vitro* essential genes. HMM was only applied on the output read counts, which included all genes with zero read counts in the input pool. Some of the genes identified ES with HMM were those with zero read counts in the input pool, so they are differentiated as *in vitro* essentials. However, other genes with zero read counts in the input pool were not identified to be essential genes by HMM based on the number of TA sites and the state of adjacent genes.249 coding regions with zero reads in inoculum were found to be essential for *in vitro* survival, whereas 42 genes were identified with an *in vitro* growth-defect state (Supplementary Tables 5, 6 in Supplementary material 1). Over 72% of orthologues of these MAP genes are also *in vitro* essential regions in *M. tuberculosis* H37Rv. Gene ontology enrichment analysis of these genes revealed a high representation of genes involved in intermediary metabolism and respiration (47.6%), information pathway (22.2%), cell wall and cell processing (17.1%).

### Essentiality of unannotated regions among intergenic regions

Apart from annotated features, the HMM has the benefit of identifying the impact that intergenic regions may have on growth. Despite little knowledge about them, regulatory functions are attributed to them. For example, non-coding RNAs (tRNA, rRNA, sRNA) that are yet to be identified may exist in intergenic regions. Moreover, intergenic regions may contain stress response regulators such as MprAB (Pang et al., 2011, 2013), and DNA sequences that act as gene promoters and enhancers (Schmidt et al., 2015). Some intergenic regions of *M. tuberculosis* even exhibit expression signals (Fu and Shinnick, 2007). A total of 251 unannotated regions were identified as essential, whereas 188 were associated with growth defect status (Supplementary Tables 10–13 in Supplementary material 1). Within the intergenic region between MAP3694c and MAP3695, a large segment of 10 sites with 5 TA mutants was determined to be essential. MAP3694c encodes for fadE5 (an acyl-CoA dehydrogenase), whereas MAP3695 is a hypothetical gene and was found to be a non-essential gene for *in vitro* growth.

### Gene ontology enrichment analysis

**FIGURE 3:**
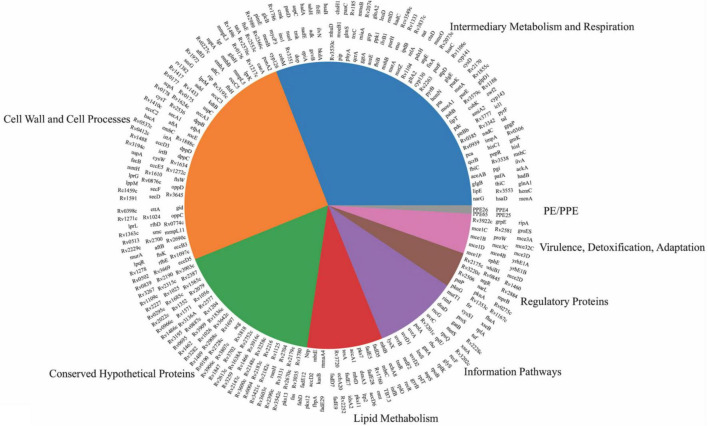
Distribution of *in vivo* essential and growth-defect genes over eight functional categories. Genes mentioned on top of each pie are from that specific category.

Gene ontology (GO) enrichment analysis on both sets of *in vivo* essential and growth-defect genes, revealed an overrepresentation of genes for biological process, molecular function, and chemical component categories (Supplementary Table 14 in Supplementary material 1 and Supplementary Figures 1–5 in Supplementary material 2). Over 84% of the biological process genes are related to cellular and metabolic processes. Over 78% of molecular functions are related to binding and catalyst activities. Over 86% of cellular components fall into the cellular anatomical entity category. More than 60% of proteins belong to the metabolite interconversion enzyme class. Significant GO terms for the top-ranked genes demonstrate the involvement of the putative *in vivo* essential genes in DNA repair, pathogenesis, symbiosis and parasitism, growth, response to stimuli and oxidation-reduction processes. Orthologues of the essential genes were distributed in 44 pathways in *M. tuberculosis*. By function prediction, 66% of *in vivo* essential and growth defect genes were categorized into intermediary metabolism and respiration, cell wall and cell processing, and information pathways, which are fundamental functions that exist in most bacteria species. Genes encoding hypothetical proteins were identified as constituting 16% of genes, whereas 18% of genes were functionally categorized into virulence/detoxification/adaptation, lipid metabolism, regulatory proteins, and PE/PPE proteins (Figure 3).

## Discussion

To study gene essentiality on a genome-wide scale, a transposon mutant pool (input) was selected on kanamycin plates (*in vitro* selection) and subjected to *in vivo* selection (output) by isolation from calf intestinal tissue after experimental oral infection. Illumina high-throughput sequencing technology was used to identify the transposon insertion sites and read counts at each site in the input and output pool. They were analyzed to determine the genes essential for MAP survival inside a ruminant natural host. Our data revealed that identified *in vivo* essential genes are mostly involved in MAP growth, symbiosis and parasitism, response to stimulus, oxidation-reduction process, pathogenesis and DNA repair; 64% of these genes having orthologues in *M. tuberculosis* were also identified as essential genes.

**FIGURE 4:**
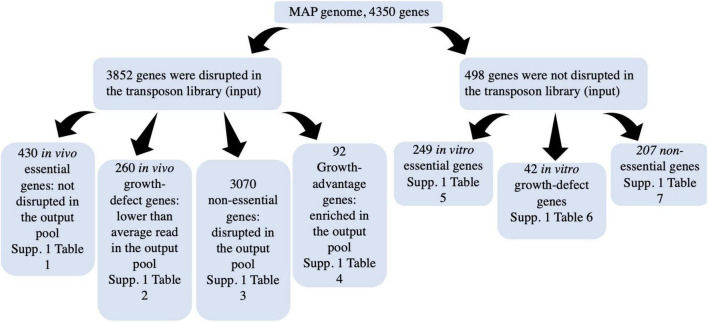
Distribution of 4,350 genes of MAP genome into different categories.

In a study using Tn-seq to study MAP survival albeit in a mouse model, the inoculum produced by Wang et al. (2014) successfully interrupted 24% of the TA dinucleotide sites and 56% of the genes in the MAP K-10 genome. The MAP A1-157 pooled library used in the present study had significantly more coverage. Our inoculum (input pool) successfully represented 56% of the TA dinucleotide sites and 88% of the genes in the A1-157 genome. This is likely due to the combination of three independent libraries to maximally saturate the number of transposon insertions. The ability to represent more TA dinucleotide sites provided the opportunity to analyze more potential essential genes and increased our confidence in identifying virulence genes since multiple TA sites per gene could be analyzed in parallel. In addition, MAP A1-157 was isolated from a clinical Johne’s disease cow whereas MAP K-10 is a recognized laboratory strain with limited virulence (Fernández et al., 2014; Stevenson, 2015). Each calf received an inoculum in which most of the mutants had at least 10^6^ CFUs present which is considered the minimum infectious dose for MAP (Hines et al., 2007). This suggests that most of the mutants were administered at an infectious level, and their absence from the cultured tissues was not a reflection of too few mutants available for infection (Figure 4 and Supplementary Tables 3, 4, 7). Representing 100% of the MAP genome with mutant strains is unlikely since some mutations will have deleterious effects on *in vitro* survival. Over 50% of undisrupted genes in the input pool are found to be essential for *in vitro* growth of MAP including genes associated with intermediary metabolism (47%), information pathways (22%), and cell wall processing (17%). Genes coding for PurC, PurL, and PurN are essential for *in vitro* growth and play role in purine biosynthesis. These genes were also found to be essential for *in vitro* growth of H37Rv by analysis of saturated Himar1 transposon libraries (Griffin et al., 2011; DeJesus et al., 2017). From the intermediary metabolism category, genes involved in histidine, aromatic amino acids, ATP, protoheme, riboflavin, arginine, and FOLIC acid biosynthesis are also found to be essential for *in vitro* growth of MAP and H37Rv (Griffin et al., 2011; DeJesus et al., 2017; Minato et al., 2019). *In vitro* essential genes from the information pathway category included RNA polymerase sigma factor sigma A (sigA), a transcription initiation factor in bacteria that is a housekeeping regulatory gene active during the exponential phase and facilitates the specific binding of RNA polymerase to gene promoters (Burgess, 2021). SigA was also found to be essential for *in vitro* growth of H37Rv. Upregulation of sigA promoted the growth of recombinant *M. tuberculosis* in macrophages and mice lungs (Wu et al., 2004). From the cell wall category, genes coding ESAT-6-like proteins, lipopolysaccharide and peptidoglycan biosynthesis are found to be essential for *in vitro* growth of MAP.

To remove potential mutants that would not be capable of growing *in vitro*, the initial steps in developing and selecting the mutants involve growth on solid kanamycin media in which the colonies were scraped and pooled. The input pool was used to examine potential virulence genes essential for infecting the intestinal tissues of dairy cattle. This method uses massive parallel sequencing of short reads of millions of DNA strands which provided a detailed picture of the mutants present in the initial inoculum (input) and the mutants that were recovered from the tissues from 5 infected calves at 2 months PI (output). Compared to the genes reported by Wang et al. (2014), of all 415 genes essential in MAP K-10, 14% (59) were found to be essential and growth-defect in MAP A1-157. This could be due to different evaluation methods. Wang et al. (2014) did not evaluate the genes by compiling all TA sites together; instead, the genes were reported by each TA site, and they included all TA sites with greater than 1 log (10 reads) in the inoculum. Evaluating the data by TA sites only may have resulted in false-positive genes being reported as possible essential genes (Wang et al., 2014). Therefore, we cannot make any conclusions regarding common essential virulent MAP genes in both mice and dairy calf models.

For further evaluation of our results, we have compared our results to those obtained by Griffin et al. (2011) by calculating p-values for each gene through the following equation:


(1) $$p\;\left(k\right)=1-\text{exp }\left[-\exp\left(\frac{\mu \;-\;k}{\sigma }\right)\right]$$

in which μ and σ are the statistics of the longest run for the gene based on the extreme value distribution given in materials and methods section. The related calculations including loci, total TA length, total insertions, expected run of unoccupied sites, maximum run of unoccupied sites, p-values and the state of each gene was provided in Supplementary Table 15 of Supplementary material 1. Our results correlate well with the results obtained by Griffin et al. (2011) Over 89% of genes we found essential by HMM are also found essential employing Griffin’s method and the average p-value of genes which were identified as essential in our HMM analysis, were 4.1% which is comparable to the 5% p-value threshold employed by Griffin et al. (2011) Additionally, the average p-value of genes which were identified as growth-defect, non-essential and growth-advantage in our HMM analysis were 15.2, 38.8, and 55.9% which show an ascending order consistent with the fact that the p value is proportional to the non-essentiality of genes.

In HMM, each TA site has its own state, and each TA site plays a role to conclude a global state of a gene. This resulted in many genes with a high probability of being significant for the survival of MAP in dairy cattle. Considering a global state for each gene, using extreme value distribution analysis in the present study, provides further protection from reporting false positives. The HMM can be run on one set of reads (output) and by knowing the inoculum (input) composition, *in vitro* essential genes can be separated from *in vivo* essential genes. The effect of adjacent genes states is considered by HMM and is considered to have biological basis in co-expression of neighboring genes in response to genetic and environmental variations. There is a correlation between physical vicinity in the genome, together with similar transcription factor regulation of nearby genes, with paired effects (Van Dyke et al., 2021). The HMM also enables identifying the state of non-coding unannotated regions.

### Metabolism and biosynthesis

Bacteria with mutations in genes associated with lipid metabolism, intermediary metabolism and respiration were significantly reduced in the calf tissues. Among the most essential genes with a high number of TA mutants absent from the calf tissues was *aceAb* or isocitrate lyase (ICL; MAP1643). Adenosine triphosphate (ATP) is essential intracellular chemical energy. Isocitrate lyase (*aceAb*), is associated with replenishing intermediates for the Krebs cycle (Mukhopadhyay and Purwantini, 2000) which generates ATP through the oxidation of acetyl-CoA. It has also been suggested that for *M. tuberculosis* to maintain a quiescent or non-replicating state that ICL must be upregulated and mediate energy metabolism (Wayne and Lin, 1982). MAP can remain in its primary host for 3–5 years before signs of the clinical disease manifest. While the bacteria continue to replicate and shed in the feces, they maintain a steady state within the host for a considerable amount of time. ICL was shown to be necessary for the survival of *M. tuberculosis* in a latent TB model (Betts et al., 2002; Muñoz-Elías and McKinney, 2005). It is possible that ICL maintains a similar mechanism for MAP to persist in the intestinal tract of dairy cattle, however, there is currently no published research investigating its role in MAP survival. Moreover, ICL contributes to the development of resistance against antibacterial drugs for *M. tuberculosis.* Three anti-tuberculosis drugs (isoniazid, rifampicin, and streptomycin) trigger ICL in *M. tuberculosis*. ICL-deficient *M. tuberculosis* is much more susceptible to these 3 antibiotics suggesting that ICL plays a role in antibiotic resistance (Nandakumar et al., 2014).

Pyruvate carboxylase (MAP0294c) with 11 TA mutants, is also an important enzyme for producing intermediates of the TCA cycle, and it is found to be essential *in vivo*. Pyruvate carboxylase is highly homologous in *M. smegmatis* and *M. tuberculosis*. Similar to ICL it is suggested that the anaplerotic or intermediate reactions produced by pyruvate carboxylase play a unique function during intracellular survival of *M. tuberculosis* (Basu et al., 2018). This suggests that further investigation into the enzymes required to produce TCA cycle intermediates could elucidate their role in MAP survival.

Among genes involved in intermediary metabolism, Probable GTP pyrophosphokinase *relA* (MAP1047) was also found to be essential *in vivo*. This enzyme coordinates the adaptation of cellular activities to nutritional conditions by catalyzing the formation of PPPGPP. Disruption in *relA* gene in H37Rv showed attenuation in mice (Dahl et al., 2003) and growth defect in THP-1 macrophages (Primm et al., 2000). MAP *relA* mutant was previously tested in bovine macrophage, goat, and calf infection model as a live-attenuated vaccine. However, while the *relA* mutant was cleared from the intestinal and associated tissue collected from the calves, it did not prevent the colonization of the challenge strain MAP K-10 (Park et al., 2011).

### Cell wall and cell processes

ATP-Binding Cassette transporters consist of a family of integral membrane proteins that includes importers and exporters. Both types can play significant roles in pathogenesis, virulence, drug efflux and survival of bacteria. For example, the two ABC importers IrtAB and BacA are involved in siderophore carboxymycobactin and vitamin B12 (hydrophilic compounds) import, respectively (Cassio Barreto de Oliveira and Balan, 2020). Mutation in MAP1531gene, coding ATP-binding protein ABC transporter bacA, impairs the growth of the bacteria in the host body. This gene was identified as growth defect gene by HMM. The mutant of bacA was found to be non-essential for *in vitro* growth of H37Rv (Griffin et al., 2011). While the growth of *M. tuberculosis* H37Rv bacA| Rv1819c mutant in B6D2/F1 mice was comparable to wild type, mice infected with mutant survive longer, suggesting that a mutant strain of Rv1819c is incapable of maintaining chronic infection in a murine model (Domenech et al., 2009). MAP2414c and MAP2413c were also found to be essential by HMM and they encode IrtA and IrtB, respectively, and are parts of IrtAB siderophores transporter. The *M. tuberculosis* mutant lacking IrtAB showed low growth rate in iron-deficient conditions, in human macrophages (Rodriguez and Smith, 2006), and in the murine lung (Griffin et al., 2011).

Several genes involved in mycobacterial growth and division were also found to be essential for *in vivo* survival, such as MAP0392c and MAP2939c, coding membrane-associated penicillin-binding protein A2 (ponA2) and peptidoglycan endopeptidase RipA, respectively. PonA localizes to the *Mycobacterium* poles and septum and regulates cell length by interacting with RipA and PknB (Maitra et al., 2019). Disruption of ponA2 provides a growth advantage for *in vitro* growth of H37Rv, while it is required for survival in primary murine macrophages (Rengarajan et al., 2005). In addition, three genes from the ftsXE system, ABC transporter implicated in cell division and peptidoglycan cleavage, were found to be essential *in vivo*: MAP1898c (ftsW), MAP2859c (ftsK), and MAP3172c (ftsE).

Some other essential genes depleted from the output pool are involved in cell wall synthesis and bioprocessing. MAP4116c gene coding Methoxy Mycolic Acid Synthase 4 (MmaA4) and MAP1968c gene coding tripeptidyl-Serine Peptidase, proteins involved in mycolic acid synthesis and cell wall biosynthesis, respectively, were identified as essential for *in vivo* survival. The mRNA levels of both genes (identified by real-time quantitative RT-PCR) increased 24 and 72 h after cultured macrophage infection (Rengarajan et al., 2005). Disruption of a homologous gene of serin peptidase and MMA4 provides a growth advantage for *in vitro* growth of H37Rv, by analysis of saturated Himar1 transposon libraries (Lamichhane et al., 2003; Sassetti et al., 2003; DeJesus et al., 2017; Minato et al., 2019). Serin peptidase is also required for growth in C57BL/6J mouse spleen and for survival in primary murine macrophages, by transposon site hybridization (TraSH) in H37Rv (Sassetti et al., 2003; Rengarajan et al., 2005). MmaA4 mutant has not yet been studied *in vivo*.

Gene coding for lipoprotein signal peptidase was also identified to be essential in our calf model. This protein specifically catalyzes the removal of signal peptides from prolipoproteins and has a role in cell wall processing. Disruption of this gene results in a growth defect of H37Rv *in vitro*, as shown by analysis of saturated Himar1 transposon libraries (Sassetti et al., 2003; Griffin et al., 2011; DeJesus et al., 2017).

### Virulence and host-pathogen interactions

Five mce coding genes (MAP3606-3610) and a possible mce-family lipoprotein (lprk) were significantly reduced from the cultured calf tissues and identified essential by HMM. The mammalian cell entry (mce) proteins are essential for the persistence and virulence of *M. tuberculosis* in the host (Forrellad et al., 2013). While the genes exist in several different bacterial species, only in mycobacteria do the *mce* genes act as an operon. The MAP genome contains eight *mce* operons which encode 74 different mce proteins (Timms et al., 2015). Interestingly, Mce3C binds to ß2 integrin and facilitates adherence and entry into macrophages (Zhang et al., 2018). Once inside the macrophage, Mce3E inhibits ERK1/2 pathway, suppressing the production of host cytokines and facilitating *M. tuberculosis* survival (Li et al., 2015).

The 4 PPE family proteins found to be essential (PPE4, PPE65) and growth defect genes (PPE25, PPE26) are members of the ESX-5 system which is associated with virulence in *M. tuberculosis*. ESX-5 is the only Type VII secretion system present in slow-growing mycobacterial species (Abdallah et al., 2009) and has been suggested to be associated with pathogenesis and virulence in *M. tuberculosis* (Gey et al., 2006). ESX-5 is involved in the secretion of many proteins which contain PE (Proline-Glutamic acid) and PPE sequences. *Mycobacterium marinum* ESX-5 mutants are unable to modulate the cytokine response in human macrophages (Abdallah et al., 2008). The PPE proteins are associated with virulence in *M. tuberculosis* (Mi et al., 2017). Expression of PPE25 and PPE26 in non-pathogenic *M. smegmatis* resulted in enhanced survival in macrophages and persistence in mouse tissues. Secretion of PPE proteins in *M. tuberculosis* modulate host immune responses through interaction with TLR4 (Choi et al., 2019). Some PPE proteins can facilitate cell resistance to surface stresses, low PH, and antibiotics to enhance intracellular survival (Gong et al., 2019) and some PE proteins decrease the rate of apoptosis in macrophages (Deng et al., 2017). There are 36 PPE orthologues between the H37Rv strain of *M. tuberculosis* and the MAP K-10 genome; however, there are no reported studies that have examined their role in MAP (Gey et al., 2006). PPE26 was previously detected by mass spectrometry and confirmed to be expressed on the cell surface of MAP(Newton et al., 2009). While very little is known about the 4 PPE proteins absent in the current trial, they may play an important role in pathogenesis. Reducing or eliminating their ability to be secreted by MAP may have significantly hindered its survival in the dairy calves. Some of the genes coding the building blocks of ESX-5 membrane complex were also found to be essential, including eccC3, eccC5, eccA3, eccB3, eccD2, eccD3, eccD5, eccE5, efpA. Additionally, eccC5 and eccB5 were also found to be vital for *M. tuberculosis* growth in THP-1 macrophages (Di Luca et al., 2012).

### Information pathway

Over 10% of *in vivo* essential genes fall into the information pathways functional category. These *in vivo* essential genes included the UvrABC incision complex that is vital for DNA repair, especially in stress conditions caused for instance by radiation exposure and oxidative damage. Genes involved in coding RecR and RecG also appeared to be essential for *in vivo* survival of MAP. RecR and RecG are ATP-dependent DNA helicase and play critical role in recombination and DNA repair. Genes involved in translational apparatus including Glu-ADT AB, probable transcription termination factor rho and ribosome releasing factor (RRF) are found to be *in vivo* essential. RFF is responsible for recycling ribosome from one round of translation to another and promotes the efficiency of translation. This gene was also found to be essential for *in vitro* growth of H37Rv (Griffin et al., 2011; DeJesus et al., 2017).

Although most of intergenic regions have not been studied and their function is not defined, the essential intergenic region between MAP3694C and MAP3695 was among several unannotated regions where transposon insertion could result in changes in MAP morphology, impairment in siderophore production, and biofilm and clump formation (Rathnaiah et al., 2014). The mutant strain of this intergenic region was also found to be attenuated for invasion and survival in monocyte-derived macrophages (Xu et al., 2013).

The current trial had several shortcomings. To ensure adequate representation of the whole MAP genome, 100,000 CFUs were needed to be represented in the inoculum and recovered from each tissue (Murry et al., 2008; Wang et al., 2014). While the inoculum consisted of enough CFUs, not enough CFUs were recovered from each animal nor tissue. Some of the colonies on kanamycin selective plates were larger than others, potentially skewing the results, which might be due to of depletion of a growth- restricted genes. To obtain enough bacteria from each tissue type, a large amount of tissue had to be cultured. Devising an effective protocol for decontaminating the intestinal tissue of endogenous bacteria and extracting MAP from within the macrophages of the tissue was difficult to accomplish without experiencing some loss of bacteria. After culturing fresh tissue and acquiring about 100,000 CFUs, additional stored tissue was cultured. However, the tissues that were frozen at –80°C resulted in a considerably lower yield. Although tissues were individually sequenced, the sequencing reads of all tissues from all animals were pooled before running the analysis to be able to represent the MAP genome and analyse the data within the power of the study. This abolished the opportunity to identify potential sources of variation on tissue and animal level. In designing future studies this type of interaction should be considered.

Aided by the high number of bacteria each calf was inoculated with and the number of different types of mutants. It is therefore possible that some mutants were rescued by other mutants. For example, multiple mutants can be phagocytosed by one macrophage (Bannantine and Stabel, 2002; Keown et al., 2012). In such case, a fatal mutant can be rescued by another mutant that survives the harsh environment within the macrophage and prevents phagocytosis. These rescue situations can occur at any major barrier imposed by the host such as infiltration of the intestinal epithelium where a MAP mutant unable to be opsonized could migrate through the gap junctions produced by other mutants (Bannantine and Bermudez, 2013). It is unknown how often these rescue situations occur, but it could lead to false positives.

In conclusion, a high number of mutants were harvested from the pooled tissues and analyzed filling a knowledge gap regarding essential genes for *in vivo* survival in the ruminant host for the first time. However, this study has its limitations and lacks the power to identify different sources of variations on tissue and animal levels. Future studies are needed to study the effect of being genetically variable on the essentiality and virulence of different genes. Targeted WGS provided a multifaceted technique to evaluate millions of reads and reveal the relative frequency in which numbers of specific mutants occurred in tissues (output) and the inoculum (input). Furthermore, functional characterization of these promising candidates will shed light on MAP pathogenesis and offer candidates for the development of vaccines against JD.

## Materials and methods

### Bacteria and growth conditions

*mycobacterium avium* subsp. *paratuberculosis* A1-157, an isolate belonging to a dominant clade containing more than 80% of all Canadian MAP isolated (Ahlstrom et al., 2015) was used as the parental strain for transposon mutant library creation. Bacteria were grown on an orbital shaker at 200 rpm at 37°C in Difco Middlebrook 7H9 media supplemented with 10% OADC (Oleic Albumin Dextrose Catalase, Becton Dickinson and Co., Sparks, MD, USA), 2 g/L mycobactin J (Allied Monitor Inc., Fayette, MO, USA) and 0.4% glycerol. Mycobacterial phage MycomarT7, containing mariner class transposon element and kanamycin resistance cassette was used for transducing the transposon into the MAP A1-157 bacterial cells (Siegrist and Rubin, 2009). Mutants were selected on Difco Middlebrook 7H11 plates containing kanamycin (50 μg/ml) and grown for 8 weeks at 37°C in Ziploc bags to prevent them from drying out.

From each library, about 5,000 colonies grew evenly on 20 plates with a diameter of 200 mm. Ahead of plating, potential clumps were broken up by passing the transduced MAP cells through a 30 g needle 10 times. A section per plate was counted after the plates were divided into 8 sections. A total of 100,000 colonies were estimated to be pooled to build one library and three libraries were combined to grow the inoculum.

### Transposon mutagenesis and inoculum library preparation

To screen for essential genes involved in the pathogenesis of MAP a library of mutants composed of disrupted genes was created using a transposon as described (Murry et al., 2008). The MycoMarT7 phagemid, which contains a kanamycin-marked transposon, was titrated, and amplified using *M. smegmatis* on kanamycin-selected plates in approximately 3 days at 30°C. Three independent broth cultures of MAP were grown to an OD_600_ of ∼1.0, approximately 2 × 10^9^ CFU of MAP A1-157 as determined by the wet weight method (Hines et al., 2007). MAP were transduced with ∼4 × 10^10^ phages (MOI = 20; Murry et al., 2008) in MP buffer (50 mM Tris-HCl [pH 7.5], 150 mM NaCl, 10 mM MgSO_4_, 2 mM CaCl_2_) for 4 h at 37°C. 7H9 medium enriched with Mycobactin J (2 g/L), glycerol (0.4%) and OADC (10%) was added to the MP buffer mixture and incubated overnight at 37°C in a shaking incubator. Mutants were spun down, resuspended in 7H9 medium, and plated on 7H11 kanamycin selective plates. After 8 weeks, kanamycin-resistant colonies were counted and scraped from the plates using a cell scraper (1.8 cm blade, BD Falcon Cell Scraper, Fisher Scientific, Pittsburgh, PA), resuspended in 7H9 medium and vigorously vortexed to break up the clumps. Three independent transposon libraries were produced and equally pooled together one day before inoculation. Each library was grown to an OD_600_ of ∼1.5, aliquoted into 25% glycerol stocks, and frozen at –80°C.

### Calves

Eight Holstein-Friesian bull calves were purchased from 6 dairy farms in Alberta, Canada, that had less than 5% within-herd prevalence of MAP based on serum ELISA recorded through Lactanet (Guelph, ON, Canada). All animal experiments followed the regulations of the Canadian Council of Animal Care and were approved by the University of Calgary Animal Committee (VSACC AC13-0060). Contact was avoided between calf and dam and the calves were collected within 24 h after birth. Calves were housed in a biosafety level 2 housing facility at the University of Calgary Veterinary Science Research Station (VSRS). Calves were individually housed in custom-built pens lined with a waterproof liner to contain all contaminated bedding and manure. VSRS personnel were trained in biosafety protocols to avoid transmitting MAP between pens and outside the facility. Animals were under veterinary care during the length of the trial. Six calves were orally inoculated with 10^11^ colony-forming units of (CFUs) transposon mutants whereas two calves served as controls and were orally inoculated with the same dose of the wildtype A1-157 strain. The calves receiving the mutant library, or the wild-type strain were euthanized at 2 months post-inoculation and necropsies were performed immediately.

### Tissue collection and bacteria extraction

Four tissues were aseptically collected from each calf: the ileum, the distal jejunum, all ileal lymph nodes, and distal jejunal lymph nodes. Intestinal samples were cut down the side connecting the mesenteric tissue and the contents were washed off the tissue under running water. All tissues were stored in whirl-pak*™* bags (Nasco, Fort Atkinson, WI, USA) with PBS and transported to the lab in an insulated container. The mucosa of the intestinal tissue was scraped and collected using glass microscope slides. After the fat was trimmed and removed from the lymph nodes, a scalpel was used to cut them into very small pieces.

Tissues were homogenized using a gentleMACS™ Dissociator (Miltenyi Biotec, Inc., San Diego, CA, USA). Up to 10 g of the scraped intestinal tissue or lymph nodes was collected and divided into 2 gentleMACS™ M tubes. Tissues were incubated for 1 h in a 0.5% Triton X-100 solution with PBS enriched with 1% Y-30 Emulsion (#A6457, Sigma Aldrich, St. Louis, MO, USA) to prevent foaming. Tissue was homogenized in the gentleMACS™ Dissociator twice using program Protein_01.01. Each tissue was combined in a sterilized bottle and PBS was used to wash out any remaining tissues in the M tubes. Tissues were centrifuged at 7,000 × *g* for 20 min and the supernatant was carefully poured off. The pellet was resuspended in 25 ml of 0.75% hexadecylpyridinium chloride (HPC) in half-strength Brain-Heart Infusion (BHI) and 10–12, 4 mm sterile glass beads. Pellets were vigorously vortexed for 2–5 min to break up the pellet. Tissues were incubated for 3 h at 37°C. Tissues were centrifuged at 7,000 × *g* for 15 min and the supernatant was poured off. The pellets were put into Para-JEM™ AS (Thermo Scientific, Waltham, MA, USA) antibiotic mixture (0.2 ml Para-JEM™ AS (Vancomycin, Nalidixic Acid, and Amphotericin), 1.5 ml full-strength BHI, 1.3 ml ddH_2_O) and incubated overnight at 37°C.

The next day 25 ml of PBS was added, the pellets vigorously vortexed to break up the tissue, and centrifuged at 4,700 × *g* for 15 min. The supernatant was poured off and the tissue resuspended in 25 ml of 0.2 M sucrose. Tissue was centrifuged at a very low speed (200 × *g* for 15 min) to allow the sucrose gradient to separate bacteria from the tissue. The supernatant was carefully pipetted, and the sucrose gradient repeated. The supernatant was spun down at 4,700 × *g* for 15 min, resuspended in PBS, and plated on several 7H11 kanamycin selective plates with Amphotericin B. The plates were incubated for 8 weeks. All the CFUs were counted and recorded. The plates were scraped using cell scrapers scraper (1.8 cm blade, BD Falcon Cell Scraper, Fisher Scientific, Pittsburgh, PA), and the bacteria were collected by individual calf tissue and frozen at –80°C.

### Deoxyribonucleic acid extraction

MAP colonies were incubated in 10 ml of 1 × TE Buffer with lysozyme (1 mg/ml; Sigma-Aldrich, St. Louis, MO, USA) in a shaking incubator for 2 days at 37°C. Then the samples were incubated for 20 min at 65°C with 10% SDS and Proteinase K (10mg/ml). To separate the lipid-rich cell wall of MAP, a CTAB/NaCL solution was added and incubated for 10 min at 65°C. Chloroform:isoamyl alcohol was added and centrifuged for 20 min at 2,000 × *g*. The upper phase was transferred to ice cold isopropanol then centrifuged at 10,000 × *g* for 10 min at 4°C. The pellet was resuspended in ice cold 70% ethanol and centrifuged for 10 min at 10,000 × *g* at 4°C. The supernatant was removed, and the DNA was resuspended in ddH_2_O.

### Transposon next-generation sequencing

The TnSeq libraries were constructed as previously described (Wang et al., 2014). Briefly, DNA was partially digested and ligated with asymmetric adapters. The DNA fragments with the Mariner transposon junction were amplified and Illumina sequencing primer sequences were added by nested PCR (Griffin et al., 2011). Amplified fragments were gel purified and fragments between 250 and 400 base pairs were selected and sequenced with a generic Illumina primer (5′ACACTCTTTCCCTACACGACGCTCTTCCGATCT) using an Illumina HiSeq2000 system at the Génome Québec Innovation Centre. One hundred base pair reads were generated. The transposon sequence (CGGGGACTT ATCAGCCAACCTGT) was trimmed using cutadapt (Martin, 2011). Reads were aligned to the MAP K-10 reference genome using Bowtie2 alignment software (Li et al., 2005). SAMtools was used to convert Aligned Sequence Alignment/Map (SAM) files into binary BAM files (Li et al., 2009). Custom scripts in MATLAB^®^ were used to analyze and map the reads to genomic TA sites. The number of reads detected, and strand orientation were determined for each TA insertion site. Each insertion site coordinate was mapped to a gene or region as region annotated in RefSeq file NC_002944.2.ptt.^1^ Read positions were visualized by either Integrative Genomics Viewer (Robinson et al., 2011; Thorvaldsdóttir et al., 2013)^2^ or DNAPlotter^3^ (Carver et al., 2009).

For calculation of CFU/mutant, the number of reads of all targeted TA sites identified in the inoculum were added up for each gene separately and divided by the total number of reads (8605962) collected from the inoculum, resulting in the portion of mutants of a specific gene compared to the total pool. The approximate number of mutants for each gene in each inoculum of 10^11^ was calculated with this proportion. Frequency histograms were calculated for all genes found mutated in the inoculum.

### The Hidden Markov Model

A Hidden Markov Model (HMM), a statistical tool used to model systems with stochastic behavior, was used to identify the essentiality of genes. In its simplest form, which is called first order HMM, it is assumed that the future state of the system is only a function of the current state and does not depend on any other older states. The employed HMM in this work is based on the HMM originally proposed by Griffin et al. (2011), that categorizes genes into four different groups including essential genes that are devoid of insertions, growth-defect genes whose disruption impairs the growth of the organism, non-essential genes whose insertion does not affect the growth of the organism, and growth-advantage genes that have metabolic costs and their insertion promote the growth of the organism. Essential, Non-Essential, Growth-Defect and Growth-Advantage define our hidden state space while the sequence of read counts at TA sites specify our observations. The initial state probabilities were set to 83, 15, 1, and 1% for the states of Essential, Non-Essential, Growth-Defect and Growth-Advantage, respectively. The initial probabilities have been set based on the expectations for bacterial organisms. However, for large sequences which is the case here, the initial probabilities do not affect the results as the HMM converges to equilibrium. Emission probability *p*(*O*_*k*_|*S*_*i*_) which expresses the probability of an observation *O*_*k*_ being generated from a state *S*_*i*_ was calculated using geometric probability distribution through the following equation:


(2) $$p\;\left(O_{k}|\;S_{i}\right)=\alpha_{i}\,{\left(1-\alpha_{i}\right)}^{O_{k}}$$

Where *O*_*k*_ and *S*_*i*_ show *k*th observation and *i*th state, respectively. α_*i*_ is a state-related parameter which have been set to $1/\bar{O}$, 0.99, $1/\left(0.01\,\bar{O}+2\right)$ and $1/5\,\bar{O}$, for the states of Essential, Non-Essential, Growth-Defect and Growth-Advantage, respectively. $\bar{O}$ is the average of non-zero read counts. The calculated emission probabilities were relegated to Supplementary Table 16 in Supplementary material 1. The probability of moving from a state to another state is represented by transmission matrix. A symmetric matrix was applied as the transmission matrix in which, the value of diagonal elements was calculated as follows:


(3) $$T_{i\,i}=1-p\;{\left(\;0|\;S_{N\,E}\right)}^{\beta }$$

where β is a parameter which have been chosen to be the minimum read count which satisfies the condition β(1−*p*_*ins*_) in which *p*_*ins*_ is the empirically calculated insertion probability obtained by regions with more than 9 consecutive TA sites without insertions. The non-diagonal elements in each row were simply set to (1−*T*_*ii*_)/3. In this particular definition of the transmission matrix, the probability of self-transition (i.e., the diagonal elements) is near 1 while the probability of any other transitions from a state to another (i.e., off-diagonal elements) is near zero, leading to a smooth transition between different states. Finally, the Viterbi algorithm was applied to identify the most likely sequence of hidden states that best explains the given sequence of observations. In this algorithm, which is a recursive dynamic programming algorithm, given that the probability of being at each state at the previous step was already computed, the probability of being at each state at the current step is computed and the path with the maximum probability determines the most likely state.

In addition to identifying the state of local TA sites, it is also necessary to identify the state of each individual genes in the genomic sequence. Genes in which all the TA sites belong to the same state, were simply classified as that state. However, there are some genes which contain regions with different states. In these genes, if the number of TA counts labeled as Essential were equal or higher than a threshold, that gene was classified as Essential, otherwise its state was considered to be the state with the maximum frequency of TA sites within that gene. The threshold is μ + 3σ in which μ and σ are the statistics of the longest run for the gene based on the extreme value distribution and are defined as described (Griffin et al., 2011):


(4) $$\mu =-\frac{\gamma \;+\;Ln\;\left[l\;\left(1\;-\;\rho \right)\right]}{L\,n\,\rho }$$

(5) $$\sigma =\frac{1}{\pi }\,\sqrt{6\;{\left(L\,n\,\rho \right)}^{2}+\frac{1}{12}}$$

Where γ = 0.5771 is Euler number, ρ is the insertion density and *l* is the region length.

### Gene ontology enrichment analysis

Enrichment analysis was done using the online tool of the Gene ontology Consortium,^4^ by uploading Mtb orthologues of the MAP genes identified by comparing genomes in https://img.jgi.doe.gov/cgi-bin/m/main.cgi.

## Data availability statement

The data presented in this study are deposited in the NCBI repository (https://www.ncbi.nlm.nih.gov/biosample), accession number PRJNA889425.

## Ethics statement

The animal study was reviewed and approved by the Veterinary Science Animal Care Committee of the University of Calgary (AC13-0060).

## Author contributions

RE designed the computational model and framework, analyzed the data, and took the lead in writing the manuscript with input from all authors. AM prepared the mutant library and carried out the animal trial and sample collection. JW provided guidance in creating the mutant libraries and performed the sequencing experiment. MB helped with experimental design and manuscript revision. HB helped with experimental design and manuscript revision. JDB acquired funds, devised the project and main conceptual ideas and manuscript outline.

## Footnotes

1
https://www.ncbi.nlm.nih.gov/nuccore/41406098

2
http://www.broadinstitute.org/igv/

3
http://www.sanger.ac.uk/resources/software/dnaplotter/

4
geneontology.org
