# Supplementary material for: Identification of essential genes in Mycobacterium avium subsp. paratuberculosis genome for persistence in dairy calves
Source: Front Microbiol. 2022 Oct 20;13:994421. doi: 10.3389/fmicb.2022.994421 (PMC9631821; doi:10.3389/fmicb.2022.994421)
Supplement: Supplementary file 2 [file Data_Sheet_2.docx]

**Supplementary Material 2:** Gene Ontology Enrichment Analysis

Figure S1: Biological process

Figure S2: Molecular function

Figure S3: Cellular components

Figure S4: Protein Classes

Figure S5: Pathways
